# Novel SrTiO_3_/NaTaO_3_ and visible-light-driven SrTiO_3_/NaTaO_3_:N nano-heterojunctions with high interface-lattice matching for efficient photocatalytic removal of organic dye

**DOI:** 10.1039/c8ra02121j

**Published:** 2018-05-24

**Authors:** Sai Wang, Xuewen Xu, Han Luo, Chaochao Cao, Xiaoyu Song, Jianling Zhao, Jun Zhang, Chengchun Tang

**Affiliations:** School of Materials Science and Engineering, Hebei University of Technology Tianjin 300130 P. R. China xuxuewen@hebut.edu.cn tangcc@hebut.edu.cn

## Abstract

SrTiO_3_/NaTaO_3_ (STO/NTO) heterojunction photocatalysts were successfully constructed by decorating NaTaO_3_ nanocubes with SrTiO_3_ nanoparticles *via* a hydrothermal method. The structure of the perovskites NaTaO_3_ and SrTiO_3_ bear some resemblance to each other, which increases the interface lattice match for promoting the migration of photogenerated carriers between the STO/NTO interfaces. In comparison to pristine NaTaO_3_ and SrTiO_3_ samples, the STO/NTO composites exhibited remarkably improved capacity for the degradation of rhodamine B (RhB) under ultraviolet (UV) light (*λ* < 400 nm) irradiation. Furthermore, the partial replacement of O^2−^ by N^3−^ in the TaO _6_ octahedron narrowed the band gap of NaTaO_3_, which significantly enhanced the photocatalytic performance of the SrTiO_3_/NaTaO_3_:N (STO/NTON) heterojunction under visible light (*λ* > 400 nm). Finally, the possible band structures of the STO/NTO and STO/NTON photocatalysts were proposed, which indicated that an n–n type heterojunction was constructed with a staggered gap for fast separation of photogenerated electron–hole pairs.

## Abstract 2

Novel SrTiO_3_/NaTaO_3_ and visible-light-driven SrTiO_3_/NaTaO_3_:N nano-heterojunctions with high interface–lattice match were synthesized for efficient photocatalytic removal of organic dye.

## Graphical abstract

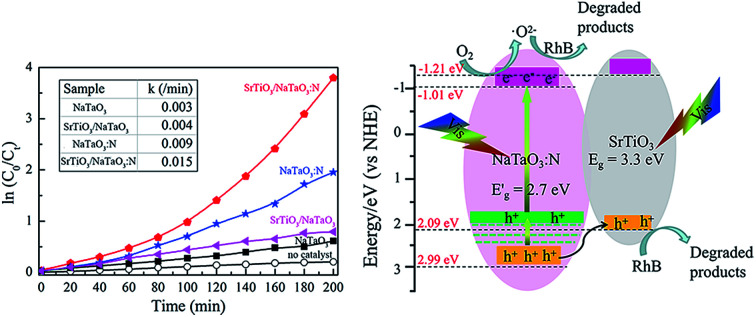

## 1. Introduction

As an important class of photocatalysts, the perovskite oxides (ABO_3_) containing a transition-metal ion with a d^0^ or d^10^ electron configuration have been widely employed in both the removal of organic pollutants and water splitting.^1–4^ In this family, NaTaO_3_ has received much attention due to its high efficiency for water splitting.^5^ However, because of its wide band gap (about 4.0 eV), NaTaO_3_ can only respond to UV light. In addition, the high recombination rate of photogenerated electron–hole pairs in bulk NaTaO_3_ usually results in a low quantum efficiency.^6,7^ Therefore, numerous approaches, including the doping of metal or non-metal elements,^8–12^ the formation of solid solutions,^13–15^ the design of various nanostructures,^16–18^ as well as forming heterojunctions,^19–27^ have been developed to improve the photocatalytic efficiency of NaTaO_3_. In previous studies, several NaTaO_3_-based composites, such as WO_3_/NaTaO_3_,^19^ Ag_2_O/NaTaO_3_,^20^ CuBi_2_O_4_/NaTaO_3_,^22^ Ta_2_O_5_/NaTaO_3_,^23^ Cu_2_O/NaTaO_3_,^24^ NiO/NaTaO_3_,^25^ Bi_2_O_3_/Bi–NaTaO_3_ and C-doped NaTaO_3_/Cl-doped TiO_2_ have been experimentally fabricated.^26,27^ Generally, these NaTaO_3_-based heterojunctions were beneficial in accelerating the charge separation, increasing the lifetime of the photogenerated carriers and improving the charge-transfer efficiency from the interface of the heterojunction to the surface of the conjoint semiconductors, and thus they exhibited superior photocatalytic activity.^28,29^ However, owing to the large lattice mismatch, it is generally difficult to fabricate NaTaO_3_-based heterojunctions with the simple oxides on a large scale.

As another extensively-studied semiconductor, the perovskite SrTiO_3_ with a cubic structure has been incorporated with numerous narrow-gap semiconductors to form visible-light driven photocatalysts.^30–34^ The lattice parameter of SrTiO_3_ is also well matched with that of NaTaO_3_.^35,36^ It is well known that there are three polymorphs of NaTaO_3_ crystallized at different temperatures, including the cubic phase at high temperature, the orthorhombic phase at room temperature, and the monoclinic phase.^37–40^ The lattice mismatch of SrTiO_3_ and cubic NaTaO_3_ is less than 1%.^41^ The unit cell of orthorhombic NaTaO_3_ is usually considered as a reconstructed structure of cubic NaTaO_3_ with the tilting of the TaO_6_ octahedra. Hence, it is structurally flexible and forms a heterojunction with SrTiO_3_ and NaTaO_3_. Theoretically, the band structure and charge carrier density of the heterojunctions of cubic NaTaO_3_ and SrTiO_3_ have been studied within the framework of density functional theory (DFT).^35,41^ The investigation of Nazir and Schwingenschlögl indicated that both p-type and n-type interfaces between cubic NaTaO_3_ and SrTiO_3_ were metallic and exhibited high charge carrier densities.^35^ Wang *et al.* reported the tunable band structure of a cubic-SrTiO_3_/cubic-NaTaO_3_ heterojunction with different layers calculated using the hybrid density functional.^41^ Experimentally, the orthorhombic NaTaO_3_ crystals have been epitaxially grown on the SrTiO_3_ (100) surface using the flux method.^36^ However, the photocatalytic performance of the heterojunction is still lacking. Furthermore, the study of the electronic structure of the heterojunction consisted of cubic SrTiO_3_ and orthorhombic NaTaO_3_ was absent.

Based on the above views, the heterojunction consisted of cubic SrTiO_3_ and orthorhombic NaTaO_3_, called STO/NTO, was synthesized in the present work *via* a hydrothermal method. The microstructure of the STO/NTO interface was studied. The as-prepared STO/NTO heterojunction exhibited high photocatalytic activity on degradation of organic dye under ultraviolet light irradiation. Furthermore, the photocatalytic performance of the visible-light driven SrTiO_3_/NaTaO_3_:N (STO/NTON) heterojunction was also investigated. The aligned band structure of the STO/NTO and STO/NTON heterojunctions were discussed.

## 2. Experimental

### 2.1. Material and sample preparation

All reagents were of analytical grade (AR) and purchased from Shanghai Aladdin Chemical Reagents Co. Ltd. They were used without further purification in the experiments.

The SrTiO_3_ powders (STO) were prepared by a hydrothermal method.^34^ In a typical synthesis, 10 mmol Ti(C_4_H_9_O)_4_ was added to 20 mL of ethylene glycol (EG) with continuous stirring to form a transparent solution. Then 20 mL of 0.5 M Sr(NO_3_)_2_ aqueous solution was added dropwise into the mixed solution. Subsequently, 10 mL of 5 M NaOH was added. The mixture was stirred for 30 min, and then transferred into a Teflon-lined autoclave and reacted at 200 °C for 24 h. Finally, the white precipitates were washed and dried to obtain STO powders.

The STO/NTO nanocomposites were also synthesized *via* a hydrothermal method. In brief, 0.035 g of the STO powders were dispersed into 60 mL of deionized water with ultrasonic treatment. Then, 24 g of NaOH and 0.3314 g of Ta_2_O_5_ were subsequently dissolved in the suspensions with vigorous stirring for 6 h. After that, the mixed solution was added into a Teflon-lined autoclave and reacted at 180 °C for 12 h. The resulting precipitates were collected and washed with deionized water several times. The final products were dried at 80 °C for 24 h before further characterization. The as-prepared samples with molar ratios of STO to NTO = 0.125, 0.25, 0.50 and 1.00 were denoted as SN-0.125, SN-0.25, SN-0.5 and SN-1, respectively.

To prepare the STO/NTON heterojunction, Ta_2_O_5_ powder was firstly heated at 950 °C for 3 h under an NH_3_ atmosphere with a flow rate of ∼100 mL min^−1^. Then, the nitrided product and NaOH powder were added to the SrTiO_3_ suspension with a Ti/Ta molar ratio of 0.25. After vigorous stirring, the mixed solution was hydrothermally reacted at 200 °C for 12 h to synthesize the STO/NTON sample.

### 2.2. Characterization

The phase composition of the as-synthesized powders was analyzed by X-ray diffractometry (XRD, Cu Kα, D8, Bruker, Germany) with a scan rate of 12° min^−1^ at room temperature. The morphology of the photocatalysts was observed by a field-emission scanning electron microscope (SEM, Nova Nano SEM450, USA) and high-resolution transmission electron microscopy (HRTEM, JEM-2010FEF, Japan). X-ray photoelectron spectroscopy (XPS, ESCALAB 250Xi, ThermoFisher Scientific, USA) was used to determine the chemical bonding of the samples. The diffuse reflection spectrum (DRS) of the samples was analyzed using a UV-Vis spectrophotometer (U-3900, Hitachi, Japan). The photoluminescence (PL) emission spectra were recorded on a fluorescent spectrophotometer (F-7000, Hitachi, Japan) at room temperature using an excitation wavelength of 280 nm.

### 2.3. Photocatalytic activity measurement

The photocatalytic activity of the STO/NTO and STO/NTON composites was evaluated by the degradation of rhodamine B (RhB) under irradiation with UV light (*λ* < 400 nm) and visible light (*λ* ≥ 400 nm), respectively. A 300 W xenon lamp (CEL-HXUV300, CEAULIGH, China) was applied as the ultraviolet-light source with an energy intensity of about 150 mW cm^−2^. A cut-off filter (*λ* ≥ 400 nm) was attached to the light source to obtain visible light with an energy density of about 100 mW cm^−2^. Cooling water in a quartz cylindrical jacket round the lamp was used to keep the reaction temperature at less than 25 °C. In each experiment, 100 mg of the catalyst powders was added into 100 mL of the RhB (10 ppm) solution. Prior to irradiation, the suspensions were magnetically stirred in the dark for *ca.* 15 min to ensure the adsorption/desorption equilibrium was reached. Then 5 mL aliquots were sampled every 15 min and centrifugated to remove the photocatalyst particles. The absorbance variation of the RhB solutions was measured using a UV-Vis spectrophotometer (U-3900H). The decolorization ratios were analyzed according to the variation in the absorption band maximum located at 553 nm for RhB. The degradation efficiency (*η*) was defined as follows:

1 *η* = (*C*_0_ − *C*_*t*_)/*C*_0_ × 100%

where, *C*_0_ and *C*_*t*_ are the initial and residual concentration of the RhB aqueous solution, respectively.

### 2.4. Photoelectrochemical measurements

The light/dark short circuit photocurrent response of the NTON and STO/NTON films supported on F-doped SnO_2_-coated glass (FTO glass) was determined under sunlight irradiation (AM1.5G, CEL-S500, CEAULIGH, China) with an energy intensity around 100 mW cm^−2^. The electrodes were prepared by dispersing the powder (50 mg) in the acetone solution of PMMA (50 mL) and then spreading on the FTO glass using a spin-coating method. Photocurrents were measured on a Zennium Workstation (Zahner-Elektrik Gmbh & Co. KG, Germany) using a standard three-electrode system with a Pt sheet as the counter electrode, a Hg/Hg_2_Cl_2_ (saturated KCl) electrode as a reference and 0.5 M Na_2_SO_4_ aqueous solution (pH = 6.8) as an electrolyte.

## 3. Results and discussion

### 3.1. STO/NTO heterojunction

**Fig. 1:**
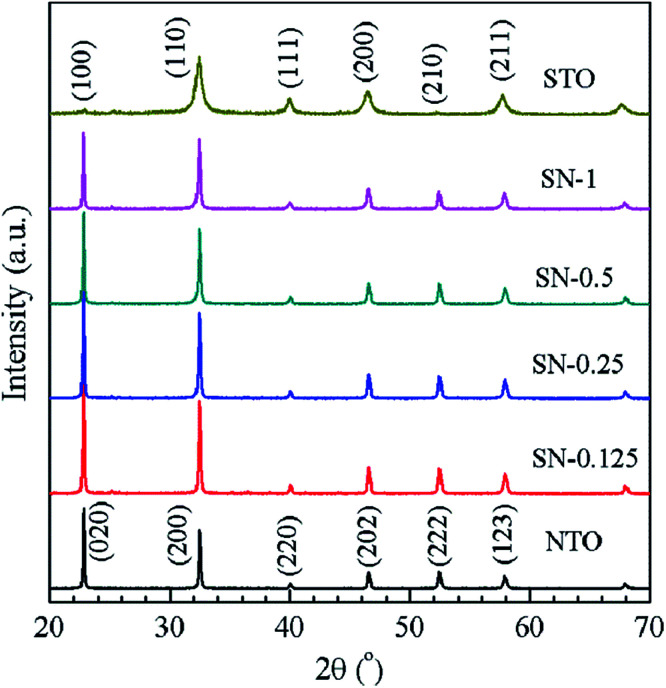
XRD patterns of the NTO and STO samples, as well as the STO/NTO nanocomposites with different molar ratios.

Typical XRD patterns are shown in Fig. 1 for NTO, STO and the four different STO/NTO samples, respectively. It is known that the XRD patterns of cubic, monoclinic and orthorhombic NaTaO_3_ are much closer to one another. According to previous research, the as-obtained NTO sample is crystallized in the orthorhombic phase (JCPDS no. 25-0863) with the space group *Pbnm* (no. 62; *a*_N_ = 5.4768 Å, *b*_N_ = 5.5212 Å, *c*_N_ = 7.7890 Å).^36,37^ The XRD pattern of the STO sample can be well indexed to cubic SrTiO_3_ (JCPDS no. 35-0734) with a lattice constant of *a*_S_ = *b*_S_ = *c*_S_ = 3.905 Å. No other peaks assigned to the impure phases can be observed in Fig. 1. Owing to their structural similarity, the position of the XRD peaks for these two perovskites is very close. Thus, it is difficult to distinguish these two compounds in the XRD patterns of the STO/NTO samples. Accordingly, it is possible to incorporate these two semiconductors into a heterojunction with less structural mismatch. The other interesting aspect of Fig. 1 is that the full-width at half-maximum (FWHM) of the diffraction peaks belonging to the STO sample is broader than that of the NTO sample. The crystal size of the STO and NTO samples was estimated to be 20 and 80 nm with Scherrer’s equation, respectively. Obviously, the size of NTO is larger than that of STO, which indicates that the growth rate of the NTO crystal is much higher than that of STO under the same hydrothermal conditions. Hence, the NTO crystals are supposed to act as the matrix of the STO/NTO nanocomposite.

**Fig. 2:**
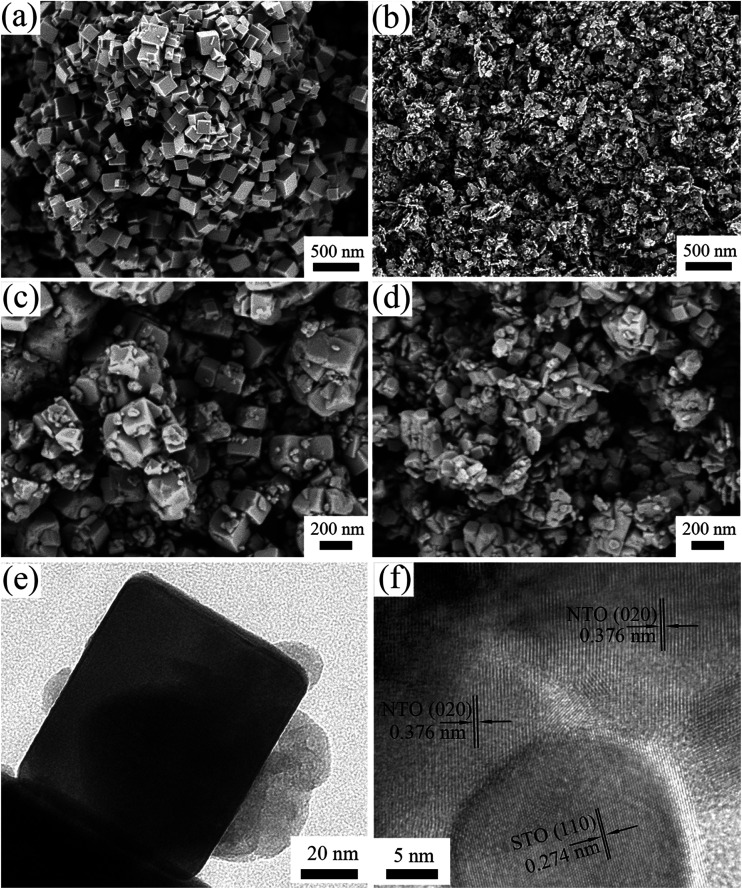
SEM images of (a) NTO, (b) STO, (c) SN-0.25 and (d) SN-0.5, and (e and f) TEM images of the SN-0.25 sample.

Fig. 2 illustrates the morphology of the NTO, STO and STO/NTO samples. The NTO sample is well-crystallized with nanocube shaped crystals with an edge length around 100 nm. The size of the STO nanoparticles is in the range 20–50 nm. For the SN-0.25 sample shown in Fig. 2(c), it is clearly seen that the SrTiO_3_ nanoparticles are uniformly coated on the facets of the NaTaO_3_ nanocubes. According to the aforementioned synthesis process, the introduced STO nanoparticles could act as nuclei for the formation of NTO. In other words, the heterogeneous nucleation of NTO occurred on the surface of the STO nanoparticles in the initial stage of the hydrothermal process. With increasing STO content in the nanocomposite, the density of the heterogeneous nuclei increased, which resulted in a gradual decrease in the size of the NTO nanocubes. However, the aggregation of both the NTO nanocubes and the STO nanoparticles was observed for the SN-0.5 sample, as shown in Fig. 2(d). Since severe aggregation is unfavorable for the separation of photogenerated carriers, the sample is anticipated to possess low photocatalytic activity.

The interface between the NTO and STO particles for the SN-0.25 sample can be clearly analyzed by TEM. As can be seen in Fig. 2(e), the SrTiO_3_ nanoparticles are intimately attached to the surface of the NaTaO_3_ nanocubes, which is consistent with the SEM results. The nanojunction structure is further determined by high resolution TEM (HRTEM) characterization, as shown in Fig. 2(f). Obviously, the SrTiO_3_ particles are in tight contact with the NaTaO_3_ matrix and form a compact heterostructure. The lattice fringes of the NaTaO_3_ cubes and SrTiO_3_ are clear, indicating the high crystallinity of the as-prepared samples. The lattice spacings of 0.376 nm and 0.274 nm can be readily assigned to the (200) crystal plane of orthorhombic NaTaO_3_ and the (110) crystal plane of cubic SrTiO_3_, respectively. As pointed out above, the relation between orthorhombic NaTaO_3_ and the cubic SrTiO_3_ cell is *a*_N_ = √2*a*_S_, *b*_N_ = √2*a*_S_, *c*_N_ = √2*a*_S_. Thus intimate contact between STO and NTO can be formed with a good lattice match, which can further promote the migration of photogenerated carriers and enhance the photocatalytic performance.

**Fig. 3:**
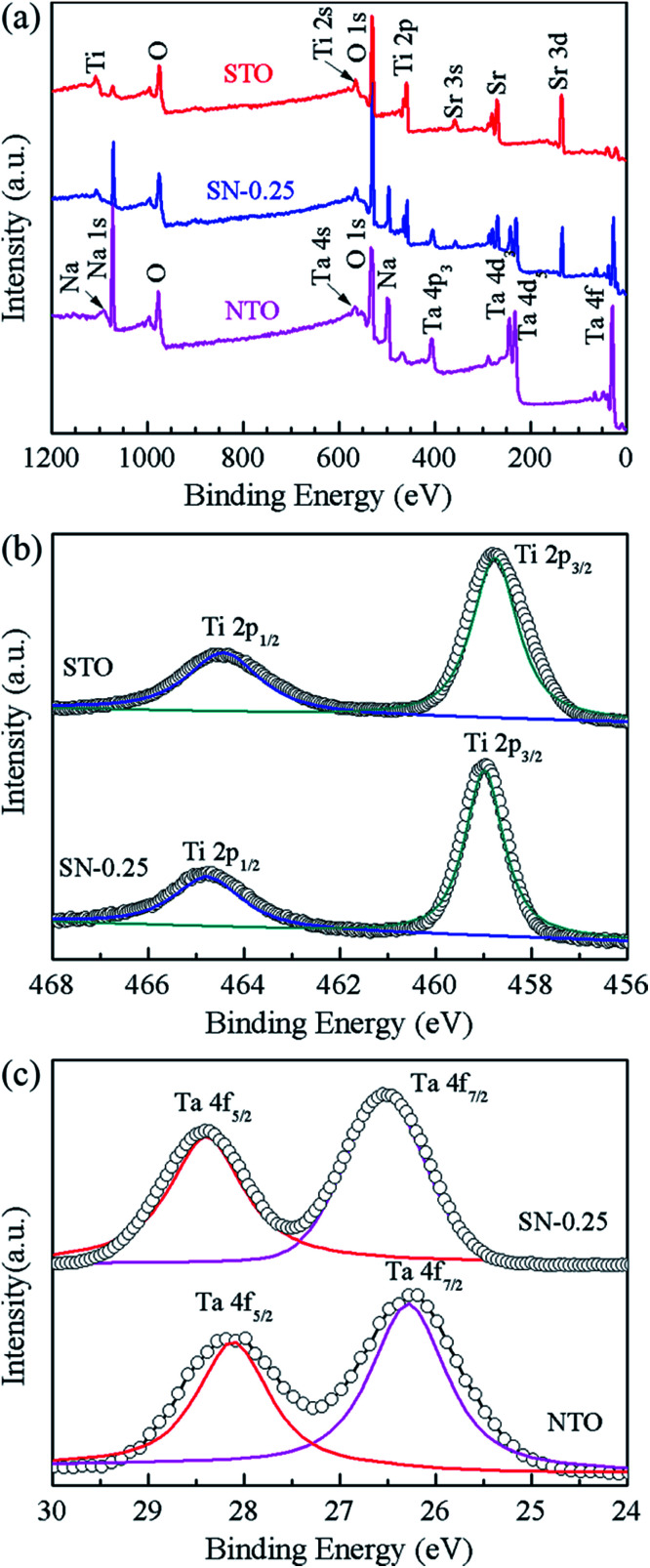
(a) The survey XPS spectra of the NTO, STO and SN-0.25 samples, (b) the high resolution spectra of Ti 2p for the STO and SN-0.25 samples, (c) the comparative spectra of the Ta 4f orbital, for the NTO and SN-0.25 samples.

XPS measurements were carried out to investigate the elemental composition and chemical state of the as-synthesized samples. Fig. 3 shows the XPS spectra of the STO, NTO and SN-0.25 samples. In the global range XPS spectrum of the SN-0.25 sample (Fig. 3(a)), the main peaks of Ta 4f, Ti 2p, Na 1s, Sr 3d and O 1s are located at 24–30, 456–468, 1070–1075, 132–138 and 528–536 eV, respectively. The XPS spectra for the STO and NTO samples are also illustrated in Fig. 3(a). The corresponding peaks are generally located in the same binding energy region with small variations. The high resolution spectra of Ti 2p for the STO and SN-0.25 samples are shown in Fig. 3(b). The two individual peaks at 458.5 and 464.5 eV for the STO sample correspond to the Ti 2p_3/2_ and Ti 2p_1/2_ spin–orbital splitting in a octahedral crystal field.^31^ After STO is coupled with NTO to form the heterojunction, these two peaks in the XPS spectrum of the SN-0.25 sample move towards higher binding energies. As shown in Fig. 3(c), a similar shift of the Ta 4f peaks, including Ta 4f_5/2_ and Ta 4f_7/2_, can also be observed. These results indicate that the chemical bonding state of the SN-0.25 surface is different to that of pristine NTO and STO. It is mainly attributed to the variation in crystal field strength after the formation of the STO/NTO heterojunction. According to the XPS results, the surface atomic molar ratios of Na/Ta, Sr/Ti, and Ti/Ta for the SN-0.25 sample are nearly close to the designed stoichiometry.

**Fig. 4:**
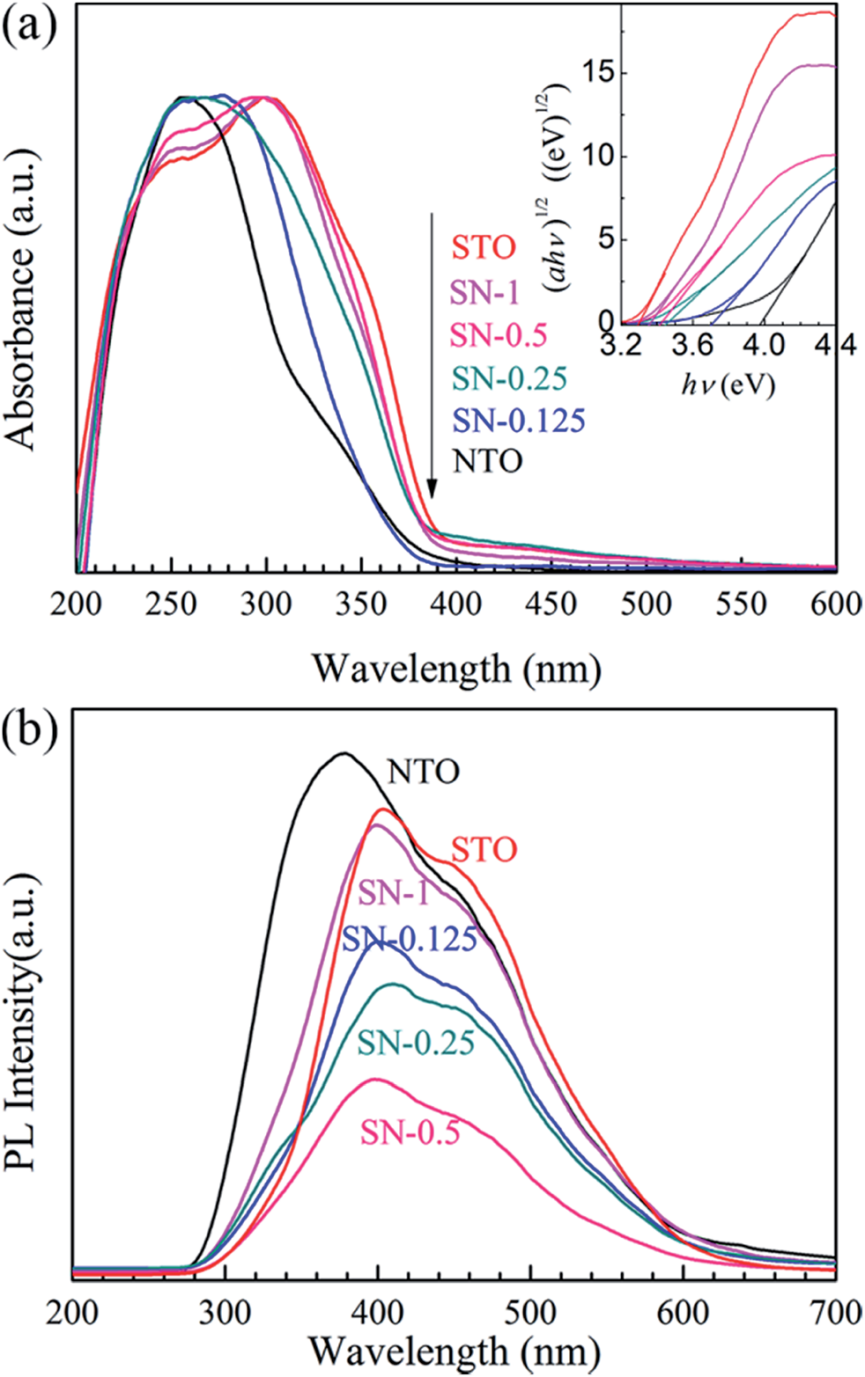
(a) UV-vis DRS of the NTO, STO and STO/NTO samples. The inset shows the curves of (*αhν*)^1/2^*vs.* photon energy (*hν*) for the photocatalysts. (b) PL emission spectra of the NTO, STO and STO/NTO samples.

The comparative UV-Vis DRS spectra of the NTO, STO and STO/NTO samples are shown in Fig. 4(a). The band gap energy (*E*_g_) for each sample is calculated from the inserted plots using the following equation,^32^

2 *αhν* = *A*(*hν* − *E*_g_)^*n*/2^

where *α*, *h*, *ν*, *E*_g_ and *A* are the absorption coefficient, the Planck constant, the light frequency, the band gap, and a constant, respectively. Since both orthorhombic NaTaO_3_ and cubic SrTiO_3_ are indirect-gap semiconductors,^42^ the value of *n* is equal to 4. As shown in Fig. 4(a), all the as-prepared samples show strong absorption in the UV light region. The band gaps of NTO and STO were estimated to be 4.0 and 3.2 eV, which are consistent with previous studies.^22,34^ With increasing STO content, the optical absorption edges of the STO/NTO samples slowly shift towards larger wavelengths, but not beyond the UV region. The band gap of the SN-0.25 sample was calculated to be 3.3 eV. Hence, the STO/NTO nano-heterojunctions can more effectively respond to UV light.

Since the PL of a semiconductor mainly results from the recombination of charge carriers, a PL emission spectrum is usually used to investigate the separation efficiency of the photoexcited electron–hole pairs in a photocatalyst. Fig. 4(b) compares the PL emission spectra for the NTO, STO and STO/NTO samples under the emission of UV light (280 nm). Obviously, the emission originating from the charge transfer of both O^2−^–Ta^5+^ and O^2−^–Ti^4+^ is efficiently suppressed by coupling the STO nanoparticles on the surface of the NTO nanocubes. It is well known that the PL emission intensity is dependent on the radiation on the surface and the structural defects of the photocatalyst.^43,44^ The higher the intensity of the emission peak, the higher the recombination rate of the photoexcited electron–hole pairs is. Thus, the recombination of the photogenerated carriers is efficiently restrained due to the formation of the STO/NTO heterojunctions, which should result in enhanced photocatalytic activity.

**Fig. 5:**
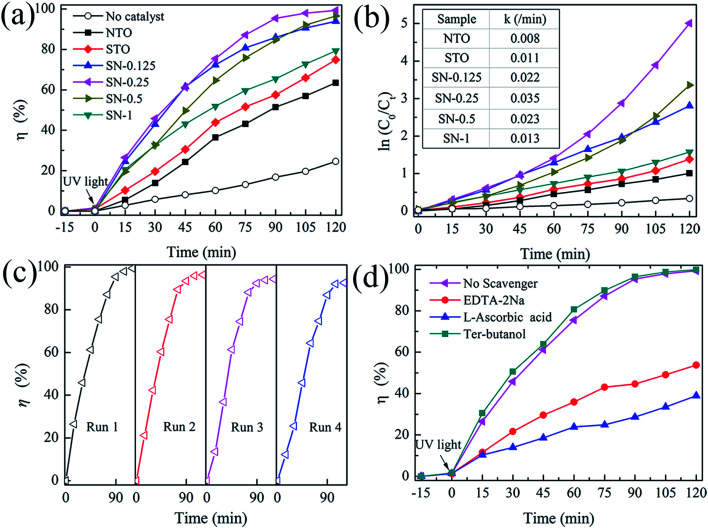
(a) Photocatalytic activity of the NTO, STO and STO/NTO samples for the degradation of RhB under UV light irradiation. (b) The pseudo-first-order reaction kinetics for RhB degradation. (c) The cyclic photocatalytic test of the SN-0.25 sample for the degradation of RhB. (d) Photocatalytic degradation of RhB by the SN-0.25 photocatalyst with different scavengers under UV irradiation.

The activity of the as-prepared photocatalysts was evaluated by the degradation of aqueous solutions of RhB as a model reaction under UV-light irradiation. Fig. 5(a) shows the decolorization of RhB by different photocatalysts under UV illumination. A blank experiment was carried out as a background check under the same conditions. Owing to the rapid recombination of the photogenerated carriers in both pristine NTO and bulk STO ,^23,33^ the degradation efficiencies of RhB for these two samples were only about 60 and 70% after 120 min irradiation with UV light, respectively. In comparison with NTO and STO, all of the STO/NTO samples exhibit superior photocatalytic activity. The SN-0.25 nanocomposite presents the highest photocatalytic activity, with a degradation efficiency of 98.2% for RhB after exposure to UV light for 105 minutes. The SN-0.5 and SN-1 samples have inferior photocatalytic activity for the degradation of RhB compared to the SN-0.25 sample, which is mainly ascribed to the high recombination rate of the electron–hole pairs in the nanoparticle aggregation as illustrated in Fig. 2(d). Moreover, for the SN-0.5 and SN-1 samples, more Sr^2+^ and Ti^4+^ ions should be incorporated into the NaTaO_3_ lattice, which results in a large amount of crystal defects acting as electron or hole traps, which then decreases the photocatalytic activity.^22,45^ As shown in Fig. 5(b), the dynamics of the degradation of RhB by the as-prepared samples generally obeys the pseudo-first-order rule. The values of the degradation rate constant, *k*, are *ca.* 0.8 × 10^−2^, 1.1 × 10^−2^ and 3.5 × 10^−2^ min^−1^ for the NTO, STO and SN-0.25 samples, respectively. Therefore, the STO/NTO heterojunction photocatalyst with an appropriate molar ratio exhibits enhanced photocatalytic activity.

The reusability of a photocatalyst is very important for its applications. As shown in Fig. 5(c), the stability of the SN-0.25 sample was confirmed by successive photocatalytic runs. The decolorization of RhB by the SN-0.25 sample shows steady performance over four cycles. The degradation rate for the forth cycle is as high as 93% after irradiation for 120 min. The deactivation of NaTaO_3_ had usually been attributed to the elution of Na^+^ on the surface of NaTaO_3_ during the photocatalytic process, which lead to the formation of surface defects, and thus accelerated the recombination of photogenerated carriers.^46^ In the present work, the SrTiO_3_ nanoparticles coated on the surface of the NaTaO_3_ nanocubes could act as a diffusion barrier for Na^+^, thus enhancing the stability of the STO/NTO heterojunction photocatalyst.

As discussed in Section 3.1, the direct recombination of electron–hole pairs is partially suppressed in STO/NTO nano-heterojunctions. However, the detailed photocatalytic mechanism is still unclear. Radical and hole trapping experiments were thus conducted to evaluate the active species during the degradation of RhB by the SN-0.25 sample. Different scavengers used as probes were introduced during the photodegradation of RhB. The scavengers included ter-butanol for hydroxyl (˙OH) radicals, l-ascorbic acid for superoxide (˙O^2−^) radicals and disodium ethylene-diaminctctraacetate (EDTA-2Na) for holes (h^+^). As shown in Fig. 5(d), the photocatalytic efficiency obviously decreases when l-ascorbic acid and EDTA-2Na were introduced under the same conditions. The introduced ter-butanol has nearly no influence on the degradation of RhB under UV irradiation. Therefore, it can be concluded that ˙O^2−^ and h^+^ radicals are the main active species for RhB photodegradation by the SN-0.25 sample, which are produced by the following reactions:^47^

3 STO/NTO + *hν* → h^+^ + e^−^ + STO/NTO

4 O_2_ + e^−^ → ˙O_2_^−^, −0.046 V *vs.* NHE

### 3.2. STO/NTON heterojunction

Although the STO/NTO samples exhibit good photocatalytic activity for the degradation of RhB, the wide band gap of these heterojunctions limits their applications. The doping of nitrogen is a usual route to tune the band structure of SrTiO_3_ or NaTaO_3_.^10^ According to previous studies, N-doped SrTiO_3_ photocatalysts were usually synthesized by a solvothermal method at 200 °C using hexamethylenetetramine (HMT) as a source of nitrogen.^48–50^ This process generally introduced excessive impurities. Thus, in the present work, the N-doped NaTaO_3_ (NTON) and SrTiO_3_/NaTaO_3_:N (STO/NTON) samples were prepared using TaON as the nitrogen source.

**Fig. 6:**
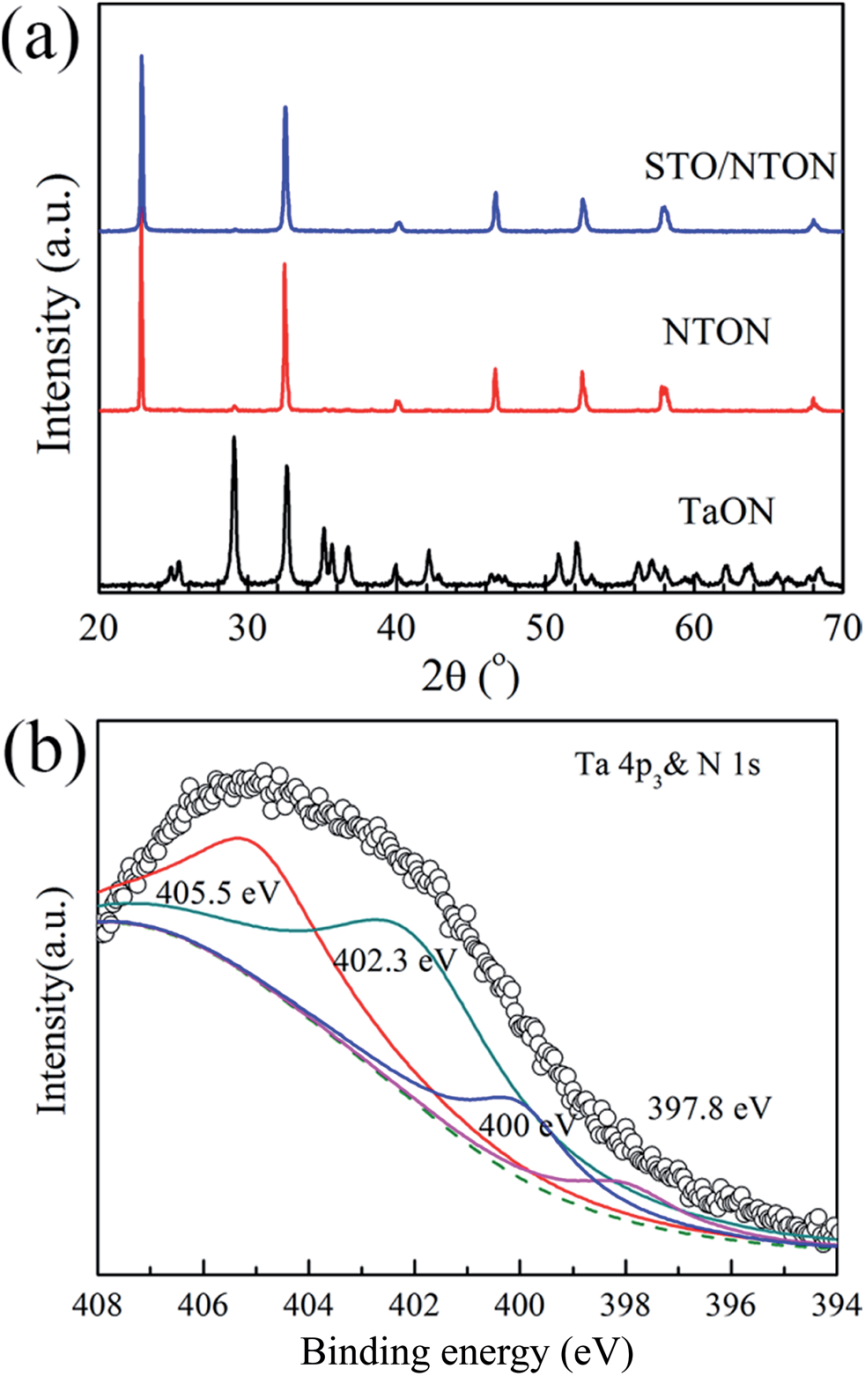
(a) XRD pattern of the as-prepared TaON, NTON and STO/NTON samples. (b) The XPS spectra of the Ta 4p_3_ & N 1s orbitals for the STO/NTON sample.

Fig. 6(a) shows the phase composition of the nitrided products of the Ta_2_O_5_, NTON and STO/NTON samples. Monolithic TaON powder was obtained by ammonifying Ta_2_O_5_ at 950 °C for 3 h. After the hydrothermal reaction, N-doped NaTaO_3_ nanocubes were formed with TaON powder as the raw material. The XRD peaks of the NTON sample can be well indexed to orthorhombic NaTaO_3_. Similar to the XRD patterns of the STO/NTO samples shown in Fig. 1, the XRD peaks of STO and NTON are highly overlapped in the STO/NTON sample. The microstructures of the as-prepared NTON and STO/NTON samples are similar to those of the NTO and SN-0.25 samples, and thus are not shown here.

To further confirm the chemical state of doped N, the XPS spectrum of the as-prepared STO/NTON sample was measured. As illustrated in Fig. 6(b), the N 1s peak can be resolved into four components centered at 397.8 eV, 400 eV, 402.3 eV and 405.5 eV, representing interstitial N-doping,^51^ anionic substitution (oxidized nitrogen),^52^ molecularly chemisorbed γ-nitrogen and the Ta 4p_3/2_ band energy,^10,52^ respectively. The results suggest the partial replacement of O^2−^ by N^3−^ at the O^2−^ site in the NTO lattice, and the formation of Ta–N bonding.^10^

**Fig. 7:**
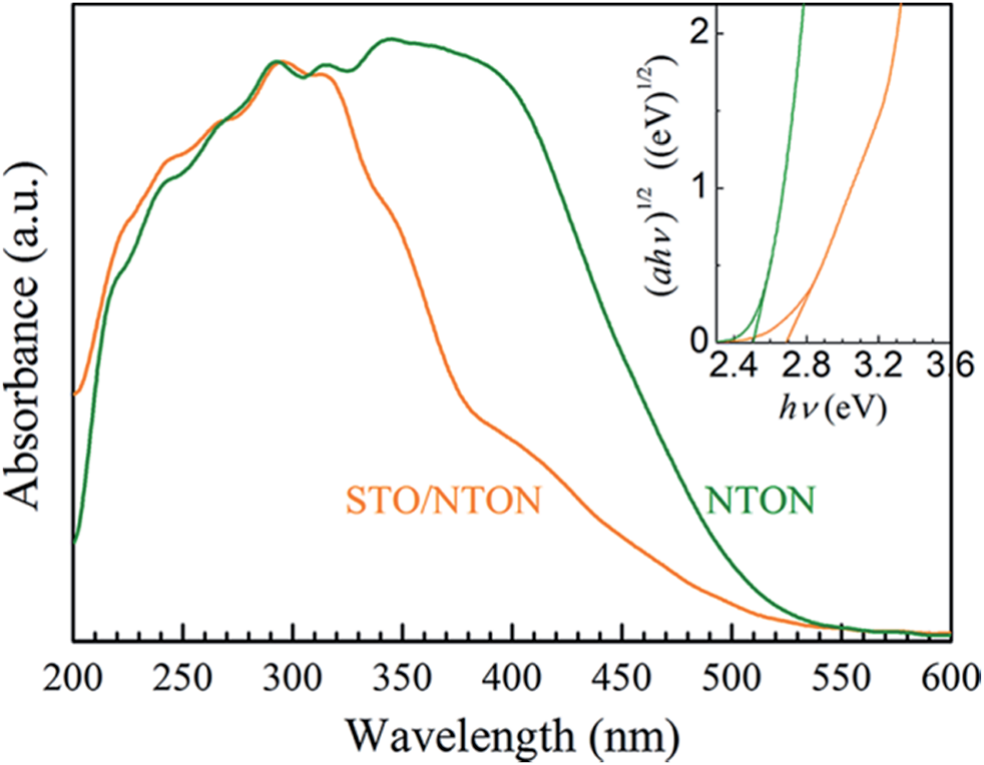
UV-vis DRS of the NTON and STO/NTON samples. The inset shows the curves of (*αhν*)^1/2^*vs.* photon energy (*hν*) for the N-doped photocatalysts.

The comparative UV-Vis DRS spectra of the NTON and STO/NTON samples are shown in Fig. 7. Obviously, the optical properties of these two samples can be effectively affected by N-doping. For the STO/NTON sample, the add-on shoulder is imposed onto the cutoff edge of the absorption spectrum, which extends the absorption range up to about 540 nm. According to eqn (2), the optical band gaps of the NTON and STO/NTON samples are estimated to be 2.5 and 2.7 eV, which are much less than those of the NTO and STO/NTO samples, respectively. The narrowed band gaps of the N-doped samples result in an effective response to visible light.

**Fig. 8:**
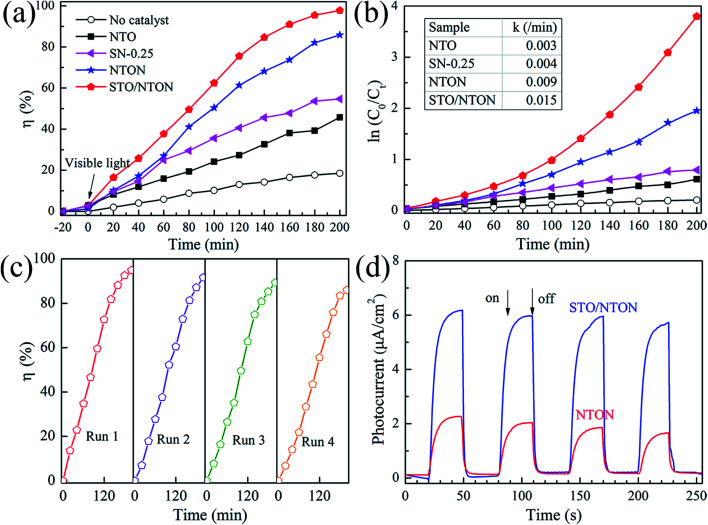
(a) Photocatalytic activity of the SN-0.25, NTON and STO/NTON samples for the degradation of RhB under visible light irradiation. (b) The pseudo-first-order reaction kinetics for RhB degradation by the NTO, SN-0.25, NTON and STO/NTON samples under visible light irradiation. (c) The cyclic photocatalytic test of the STO/NTON sample for the degradation of RhB. (d) Transient photocurrent responses of the NTON and STO/NTON samples.

The degradation of aqueous solutions of RhB was also applied to estimate the photocatalytic activity of the N-doped samples. Fig. 8(a) and (b) show the degradation curves of RhB for the NTO, SN-0.25, NTON and STO/NTON samples under visible light irradiation and the corresponding pseudo-first-order fitting curves, respectively. Obviously, because of their large band gaps, both the NTO and SN-0.25 samples exhibit poor activity for the decolorization of RhB under irradiation by visible light. The degradation efficiency of RhB for the NTON and STO/NTON samples is remarkably enhanced, which is mainly attributed to the narrowed band gaps of these two samples. Furthermore, it can be seen that the photocatalytic activity of the STO/NTON sample is much superior to that of the NTON sample. Therefore, a heterojunction should be formed between the STO and NTON nanoparticles, which can further accelerate the migration of photogenerated carriers. As shown in Fig. 8(c), the reusable stability of the STO/NTON composite was also investigated. The STO/NTON sample shows a steady performance for the degradation of RhB over four successive cycles.

To provide further evidence of the separation of photogenerated electron–hole pairs, the photocurrent responses of the NTON and STO/NTON samples over several on-off irradiation cycles at 0 V *vs.* Hg/Hg_2_Cl_2_ (saturated KCl solution) were recorded and are shown in Fig. 8(d). It is clear that the photocurrent density of these samples significantly increased and then decreased rapidly as soon as the light is turned off. The STO/NTON sample produces a much higher photocurrent density than the NTON sample, implying there is a more efficient separation of photogenerated electron–hole pairs in the heterojunction.

### 3.3. Photocatalytic mechanism

**Fig. 9:**
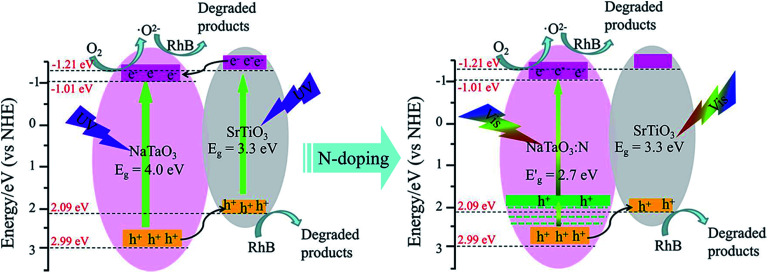
Schematic diagram for the band structure and expected charge separation of the STO/NTO and STO/NTON heterojunctions under UV and visible-light irradiation, respectively.

According to the above discussion, the energy band diagram of the STO/NTO heterojunction is supposed and illustrated in Fig. 9. Generally, the top of the valence band (VB) and the bottom of the conduction band (CB) potentials of a semiconductor, designated as *E*_CB_ and *E*_VB_, were calculated by the following equations:^53^

5 *E*_CB_ = *χ* − *E*^e^ − 0.5*E*_g_

6 *E*_VB_ = *E*_CB_ + *E*_g_

where *χ* is the absolute electronegativity of a pristine semiconductor, *E*^e^ is the energy of a free electron on the hydrogen scale (∼4.5 eV), and *E*_g_ is the band gap of a semiconductor. The values of *χ* for NaTaO_3_ and SrTiO_3_ are *ca.* 5.49 and 4.94, respectively.^22,54^ Thus, the *E*_CB_ and *E*_VB_ of orthorhombic NaTaO_3_ are estimated to be −1.01 and 2.99 eV with respect to the normal hydrogen electrode (NHE). For SrTiO_3_, the *E*_CB_ and *E*_VB_ values are calculated to be −1.21 and 2.09 eV, respectively. Since NaTaO_3_ and SrTiO_3_ are usually considered as n-type semiconductors, the Fermi levels (*E*_F_) of these two semiconductors are located close to their CB levels.^22,31^ Accordingly, the *E*_F_ of SrTiO_3_ is slightly more negative than that of NaTaO_3_. Obviously, the CB and VB levels of SrTiO_3_ are higher than the corresponding levels of NaTaO_3_, so a conventional type-II heterojunction with a staggered gap is formed between NaTaO_3_ and SrTiO_3_.^55^ As the STO/NTO sample is excited under UV light, the photogenerated electrons may simply transfer from SrTiO_3_ to NaTaO_3_, together with the migration of holes from NaTaO_3_ to SrTiO_3_. As a result, the positive and negative charges are accumulated in the VB of SrTiO_3_ and the CB of NaTaO_3_, respectively. Hence, the direct recombination of photogenerated electron–hole pairs is efficiently suppressed. Because of the strong reduction ability of the electrons in the CB of NaTaO_3_, ˙O_2_^−^ is easy to produce. However, the holes in the VB of SrTiO_3_ could not oxidize OH^−^ into ˙OH due to the low potential of the valence band maximum of SrTiO_3_. Therefore, it is easy to understand why ˙O^2−^ and h^+^ radicals are the main active species for the degradation of RhB by the SN-0.25 photocatalyst under UV irradiation.

After N doping, N 2p states are introduced above the VB of NaTaO_3_.^10^ According to the first-principle calculation results for the band structure of N-doped NaTaO_3_ and the above UV-Vis DRS for NTON, the position of the doped levels are illustrated in Fig. 9 by the green lines.^10^ The N 2p states mixed with the O 2p states result in narrowing of the band gap of the N-doped samples, which is responsible for the effective absorption of visible light. Furthermore, the photogenerated holes migrate quickly from N-doped NaTaO_3_ to SrTiO_3_ due to the lower potential energy of SrTiO_3_. Hence, a conventional type-II heterojunction is also suggested to form between the NTON and STO nanoparticles. Because of its intimate contact interface and suitable structure for the spatial separation of electron–hole pairs, the STO/NTO heterojunction and STO/NTON photocatalysts exhibit high efficiency for the degradation of RhB under UV and visible light irradiation, respectively.

## 4. Conclusions

In the present work, both the STO/NTO and visible-light-driven STO/NTO:N nano-heterojunctions were successfully synthesized by a hydrothermal method. The introduced STO nanoparticles could act as heterogeneous nuclei for the nucleation and growth of NTO. Substantial interfaces with less lattice mismatch were formed between the STO nanoparticles and the NTO nanocubes. In comparison with the pristine STO and NTO samples, the STO/NTO samples, especially the SN-0.25 sample, exhibit superior photocatalytic activity for the degradation of RhB under UV light irradiation. After nitrogen doping, the STO/NTON composite with a narrowed band gap shows enhanced photoactivity performance under visible light irradiation. With the formation of the type-II heterojunction with a staggered gap, the photogenerated electron–hole pairs are efficiently separated. The present work may offer a simple pathway to design and synthesize NaTaO_3_-based heterojunctions with high photocatalytic activity, and also provide deep insight into different heterojunction photocatalysts for practical applications.

## Conflicts of interest

There are no conflicts of interest to declare.

## Supplementary Material
